# Impact of Tool Velocity Ratio on Welding Loads and Mechanical Properties in Friction Stir‐Welded AA7075/AA2024 Plates

**DOI:** 10.1155/tswj/6123056

**Published:** 2026-01-16

**Authors:** Mutyala Rama Durga Rao, Bunga Kiran Kumar, Kiran Kumar Billa, Abhijit Bhowmik, N. Ashok

**Affiliations:** ^1^ Department of Mechanical Engineering Research Centre, Sasi Institute of Technology and Engineering, Tadepalligudem, India, sasi.ac.in; ^2^ Department of Additive Manufacturing, Mechanical Engineering, SIMATS, Saveetha Institute of Medical and Technical Sciences, Chennai, India, saveetha.com; ^3^ Centre for Research Impact and Outcome, Chitkara University, Rajpura, Punjab, India, chitkara.edu.in; ^4^ Faculty of Mechanical Engineering, Jimma Institute of Technology, Jimma, Ethiopia, ju.edu.et

**Keywords:** AA2024, AA7075, FSW, TiB_2_, tool velocity ratio

## Abstract

The emphasis on dissimilar joining of aluminum alloys has increased due to the growing need for lightweight, highly durable structures in the transportation and aerospace industries. For these applications, friction stir welding (FSW), a solid‐state joining technology that offers better structural integrity than traditional fusion techniques, has proven very successful. The force‐torque behavior and mechanical characteristics of friction stir welded dissimilar aluminum alloys, AA7075 and AA2024, with and without titanium diboride (TiB_2_) reinforcement, are investigated in this work in relation to the tool velocity ratio (*ω*/*v*). With a constant rotational speed of 1000 rpm and a 1.5° tilt angle, a cylindrical taper tool (3 mm tip, 6 mm length) was used. The traverse speeds were varied to 1, 2, 3, and 4 mm/s, yielding velocity ratios of 1000, 500, 333, and 250, respectively. To evaluate the impact of the TiB_2_ powder on joint performance, it was injected via machined grooves at the faying surfaces. The microstructural improvement, primarily grain refinement through dynamic recrystallization and Zener pinning effects from TiB_2_ particles, significantly enhanced the hardness and tensile strength of the welds. Enhanced particle dispersion and metallurgical bonding were responsible for the superior mechanical response. Because of better metallurgical bonding, grain refinement, and particle dispersion, reinforced welds demonstrated superior characteristics in microstructural, tensile, and hardness tests, particularly at higher velocity ratios (lower traverse speeds). At a velocity ratio of 1000 (1 mm/s), the reinforced samples showed the highest tensile strength (219.5 MPa), elongation (6.9%), and improved microhardness, resulting in peak joint performance. Conversely, unreinforced welds with coarser microstructures and worse mechanical properties were found at lower velocity ratios. These results provide practical advice for dissimilar alloy FSW applications in advanced engineering systems and validate that a high tool velocity ratio in conjunction with TiB_2_ reinforcement is essential for maximizing weld integrity and mechanical behavior.

## 1. Introduction

There has been a paradigm shift toward lightweight, high‐performance materials and manufacturing techniques that provide better structural integrity, fuel economy, and sustainability [1, 2]. This movement has been prompted by the expanding needs of contemporary engineering sectors, particularly aerospace, automotive, marine, and defense industries. Over the course of these improvements, the combining of aluminum alloys that are not identical to one another has been an important subject of research. The use of dissimilar joining provides one‐of‐a‐kind chances for adjusting mechanical performance and optimizing costs. This is accomplished by integrating the strengths of several base metals inside a single structural assembly. Traditional fusion‐based welding procedures, on the other hand, often encounter difficulties such as the development of brittle intermetallic compounds, high‐residual stresses, and deformation, especially in aluminum systems that are different from one another. [2–5]

**Figure 1:**
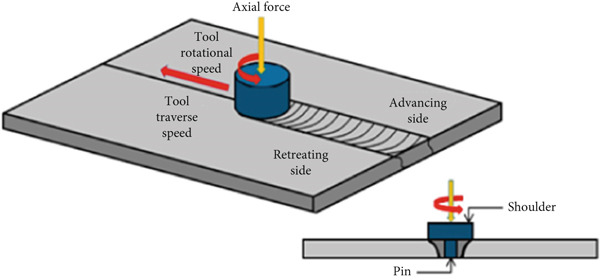
FSW process.

Friction stir welding (FSW) (Figure 1), a solid‐state joining technique that was established by The Welding Institute (TWI) in 1991, has become the preferred method for welding aluminum alloys, particularly incompatible combinations. This is because they are able to overcome the restrictions that have been mentioned above. FSW makes it possible to link materials that are far lower than their melting points. This eliminates the need for filler material and reduces the negative consequences that are associated with fusion welding [6]. The procedure employs a spinning tool that is not consumable and moves along the joint line. This tool generates frictional heat that softens the material, which in turn enables plastic deformation and stirring at the interface. A refined microstructure, higher mechanical qualities, and low flaws are all characteristics of the weld that was produced as a consequence.

When it comes to aerospace constructions, where it is necessary to strike a strategic balance between strength, fatigue resistance, and corrosion performance, the application of FSW to dissimilar aluminum alloys, such as AA7075 [7] and AA2024 [8], is especially promising. As a result of its high strength‐to‐weight ratio, the zinc‐based alloy AA7075 is frequently used. On the other hand, the copper‐based alloy AA2024 is well‐known for its exceptional fatigue resistance and machinability because of its composition. The connecting of these alloys, on the other hand, is difficult because of the variations in their chemical composition, thermal conductivity, and plastic flow behavior. These discrepancies may lead to inhomogeneous mixing and microstructural flaws. Consequently, in order to maximize the quality of the weld, it is necessary to have a comprehensive grasp of the process parameters, particularly the tool velocity ratio, which is defined as the ratio of the traverse speed to the rotational speed [9]. The tool velocity ratio has a considerable impact on the formation of heat, the flow of material, and the mixing that occurs directly at the joint contact. In contrast, a lower ratio may result in inadequate mixing or defects such as tunnel voids [10]. A larger ratio indicates that there is a greater intensity of stirring and heat input, which favors better bonding and grain refining. In recent years, researchers have investigated a variety of approaches to improve the quality of FSW joints. One of these approaches is the insertion of ceramic reinforcements such as titanium diboride (TiB_2_), which has shown promising results. The grain refiners and mechanical strengtheners that these reinforcements provide contribute to an increase in the material′s hardness and resistance to wear [11]. The purpose of this work is to explore the impact that the tool velocity ratio has on the force‐torque response and mechanical characteristics of different FSW joints formed from AA7075 and AA2024, both with and without the addition of TiB_2_ reinforcement [12]. The purpose of this study is to provide fresh insights into the dynamic relationship between velocity ratio, reinforcement, and weld performance. This will be accomplished by systematically altering the traverse speed while maintaining a constant tool rotation speed. Additionally, tensile strength, microstructure, and hardness will be analyzed. These findings have immediate repercussions for the design of structural components that are both lightweight and high‐strength, and they are intended for use in demanding industrial applications [13].

## 2. Literature Review

Since the beginning of the 21st century, the connecting of different aluminum alloys by the use of FSW has garnered a significant amount of interest from researchers. There have been a great number of studies that have shown that FSW is capable of generating sound junctions between alloys that have differing compositions and mechanical characteristics. It was Dawes and Thomas [14] who were among the first to record the possibility of FSW for connecting aluminum alloys without melting. They highlighted the advantages of FSW in terms of decreased distortion and fault generation. The researchers Elangovan and Rathinasuriyan found that the geometry of the tool and the location of the material had a substantial influence on the weld strength and the production of defects in such systems [15]. An essential metric that plays a crucial role in influencing the thermal and mechanical behavior during FSW is the tool velocity ratio, denoted as *ω*/v. In this context, *ω* represents the rotating speed of the tool, whereas v represents the traverse speed. It has a direct impact on the flow of plastic and the mixing of materials, and it regulates the amount of heat that is input per unit length of the weld.

According to Salih et al., raising the tool velocity ratio results in finer grain structures. This is because greater dynamic recrystallization rates lead to finer grain structures, which in turn improves mechanical performance [16]. Nevertheless, excessive heat input at extremely high ratios has the potential to produce grain coarsening or softening in heat‐affected zones within the material. When applied to different alloys, Trivedi et al. [17] proved that greater tool velocity ratios enhance intermixing at the interface and lower the chance of defects such as lack of penetration or kissing bonds. This was proven by the fact that the ratios of tool velocity were increased. This equilibrium is of utmost significance in combinations such as AA7075–AA2024, which have restricted metallurgical compatibility. Researchers have introduced a variety of reinforcements into the FSW zone in order to further improve joint performance. This has resulted in the creation of surface composite or hybrid joints. In order to better understand how ceramic particles like TiC, SiC, Al_2_O_3_, and TiB_2_ might improve mechanical and tribological characteristics, a significant amount of research has been conducted on these particles. Particularly noteworthy is the fact that titanium dioxide (TiO_2_) is characterized by its excellent hardness, thermal stability, and wettability with aluminum matrices. It was established by Feddal I et al. that the incorporation of nano‐TiB_2_ into aluminum FSW joints has favorable effects, which result in enhanced hardness and tensile strength [18]. In a similar manner, Ma et al. found that TiB_2_ reinforced joints exhibited improved microstructural refinement and particle dispersion [19]. Hashemi and Eghbali investigated ultrafine–grained dual‐phase low‐carbon V–Nb–Mo steel produced through severe warm rolling and inter‐critical annealing of a superferrite structure. The refined microstructure (0.95 *μ*m ferrite, 0.65 *μ*m martensite) exhibited *α* and *γ* textures, achieving 945 MPa yield strength, 1400 MPa tensile strength, and 23% elongation with ductile fracture [20]. According to Luo, axial force and torque are sensitive to changes in process parameters and may be used to forecast joint quality in real‐time [21]. This implies that these forces can be utilized to predict joint quality. When dissimilar welding is performed, force‐torque signatures often exhibit asymmetry. This is because flow characteristics vary across the weld line during the process. Clark and Ragai investigated the influence of axial force and feed rate during FSW of AA6061‐T6 using a 5‐axis CNC with adaptive torque control. Taguchi and ANOVA revealed that axial force strongly affects tensile strength, achieving up to 69% of base metal strength and optimizing weld quality at 8.3 kN and 300 mm/min [22]. In addition, reinforcement particles have the ability to enhance torque owing to their resistance to stirring, as Liu et al. pointed out in their research on reinforced aluminum joints [23]. Direct indicators of weld integrity are often used, and the mechanical characteristics of FSW joints, which include tensile strength, hardness, and elongation, are frequently observed. Wang et al. conducted research that demonstrated that different AA7075–AA2024 joints displayed maximal strength when tool offset and velocity ratios were tuned for balanced material flow [24]. In order to properly understand mechanical behavior, it is essential to conduct microstructural investigations utilizing methods like optical microscopy, scanning electron microscopy, and EBSD. These techniques show grain shape, particle dispersion, and interfacial bonding. Ceramic particle insertion greatly refined the nugget zone (NZ) and promoted high‐angle grain boundaries, which resulted in an increase in strength. This was reported by Avinash et al. [25], who carried out extensive microstructural characterization of reinforced FSW joints. This study establishes a novel correlation between the tool velocity ratio and the force‐torque response and mechanical performance of friction stir‐welded dissimilar AA7075–AA2024 joints, both with and without TiB_2_ reinforcement. The incorporation of reinforcement and systematic analysis over a range of traversal speeds provides fresh insights into the optimization of the process for structural applications that use heterogeneous materials.

## 3. Methodology

**Figure 2:**
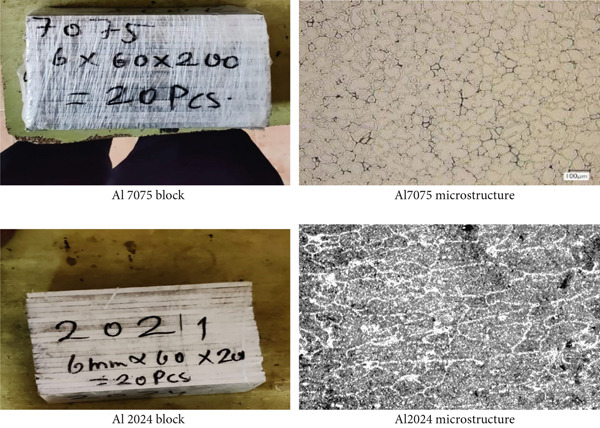
AA7075 and AA2024 Materials.

For the purpose of this investigation, the dissimilar aluminum alloys AA7075 and AA2024 (Figure 2) were chosen because of their excellent strength‐to‐weight ratios and their significance in aerospace applications.

**Figure 3:**
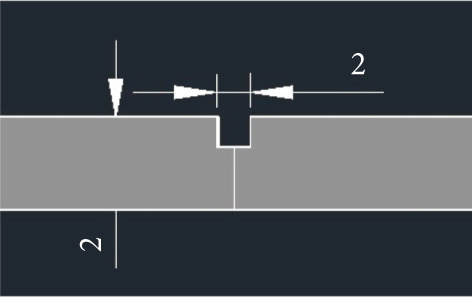
Groove in millimeters.

The metal plates were machined to dimensions of 200 mm by 60 mm by 6 mm. To include reinforcement, a groove of 200 mm in width, 1 mm in depth, and 2 mL in depth was machined (Figure 3) at the weld contact. TiB_2_, a ceramic compound that has exceptional thermal and mechanical stability, was used as the reinforcement because of the grain refining and hardening qualities that it possesses [26].

It was possible to produce eight examples, four of which had TiB_2_ reinforcement and four of which did not. The machinery utilized for the FSW process was a three–ton NC‐controlled machine that had a cylindrical taper tool with a tip diameter of three millimeters and a tip length of six millimeters. To investigate the impact of tool velocity ratio (*ω*/v), we maintained a constant rotation speed of 1000 revolutions per minute (rpm) and a tilt angle of 1.5°. Additionally, we altered traverse speeds by 1 mm/s, 2 mm/s, 3 mm/s, and 4 mm/s at different speeds.

A first step in the FSW process consisted of securing the workpieces in a clamping position and placing the tool at the interface of the butt joint. The instrument was brought into contact with the joint line and then moved down the groove at the speeds that had been specified beforehand. For the samples that were strengthened, TiB_2_ was meticulously placed into the groove before the welding process began.

**Figure 4:**
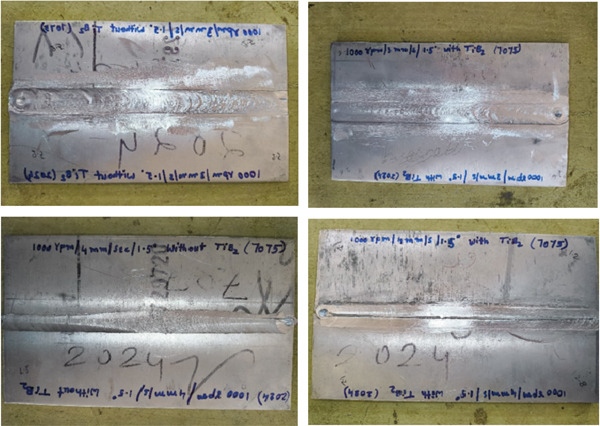
Welded specimens.

The tool velocity ratio was regulated by keeping the rotation speed while adjusting the traverse speed. This allowed for the investigation of the impacts of heat input and material flow under a variety of situations. For the purpose of avoiding residual stresses, tensile specimens were extracted using wire EDM. These specimens were then evaluated with a universal testing machine in accordance with the requirements established by ASTM E8. The Vickers micro hardness test was carried out with a load of 200 g and a dwell duration of 15 s. Keller′s reagent was used to polish and etch cross‐sectional samples from the weld area. These samples (Figure 4) were then studied under optical and scanning electron microscopes in order to investigate grain refinement, particle distribution, and the presence of defects.

## 4. Results and Discussion

### 4.1. Tensile Strength

The tensile test outcomes from this investigation highlight the significant influence of TiB_2_ reinforcement and tool traverse speed on the mechanical performance of friction stir‐welded dissimilar aluminum alloy joints. Notably, specimens reinforced with TiB_2_, consistently outperformed their unreinforced counterparts in terms of both ultimate tensile strength (UTS) and ductility. At the lowest traverse speed of 1 mm/s—corresponding to the highest tool velocity ratio—the reinforced specimen (Sample A‐2) exhibited a UTS of 219.5 MPa, which represents a measurable improvement over the 217.1 MPa observed in the unreinforced Figure 5a,b and Table 1 of sample (Sample A‐1) processed under identical conditions. Although the numerical increase may appear modest, it is technically significant when considered in conjunction with the concurrent enhancement in ductility.

Figure 5Sample‐1: 1000 rpm/1 mm/s/1.5° without TiB2. (a) Stress–strain Curve A‐1. (b) Load–displacement Curve A‐1.

**Figure:**
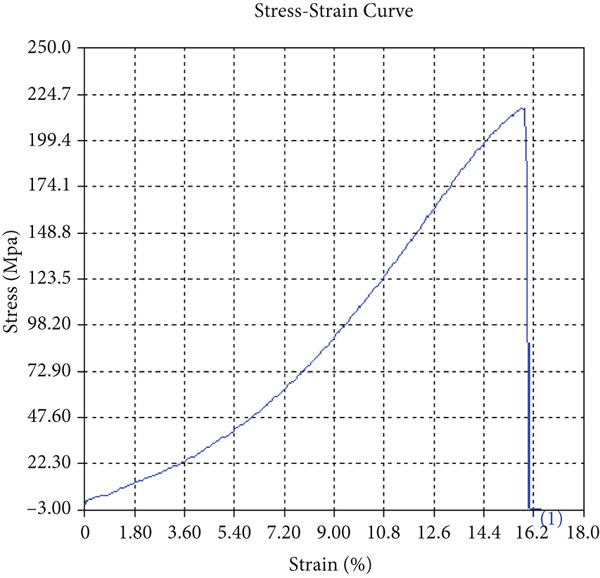
(a)

**Figure:**
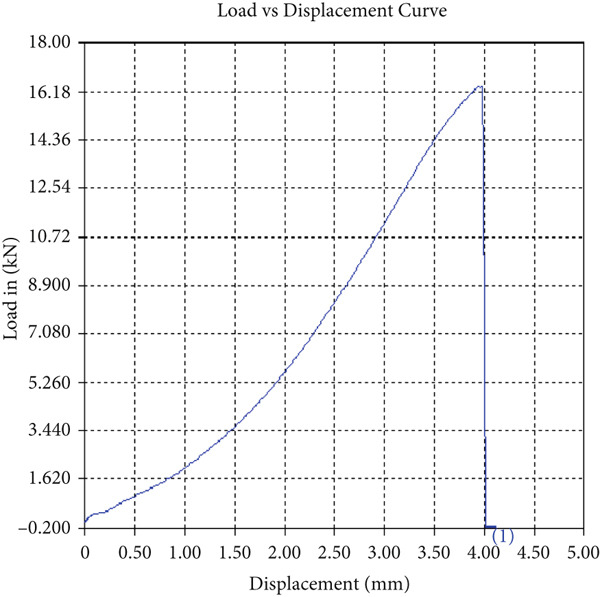
(b)

**Table 1:** Mechanical properties from tensile test of Sample A‐1.

Change in length (mm)	41.75
Maximum tensile force (kN)	16.36
Maximum yield force (kN)	13.5
Young′s modulus E (GPa)	1.41
Percentage of elongation (%)	4.375
Ultimate tensile stress (MPa)	217.1201062
Maximum yield stress (MPa)	179.1639018
0.2% Offset yield stress (MPa)	182.7471798

The findings from Figures 6a,b, 7, 8, 9, 10, 11, and 12a,b and Tables 2, 3, 4, 5, 6, 7, and 8 illustrate the tensile performance trends of AA7075–AA2024 friction stir welds at different traverse speeds, with and without TiB_2_ reinforcement. At a rate of 1 mm/s, the incorporation of TiB_2_ (Sample A‐2) enhanced elongation to 6.9% compared with the unreinforced sample, with a marginal increase in UTS to 219.5 MPa. As shown in Figure 7, the stress‐strain curve for Sample A‐3 at a transverse speed of 2mm/s demonstrates a notable decrease in tensile strength compared with the TiB2‐reinforced samples. Figure 8 highlights the stress‐strain and load displacement curves for Sample A‐4, which was processed at 2 mm/s transverse speed with TiB2, showing an improvement in tensile strength and elongation. The results presented in Table 4 indicate that TiB2 reinforcement at 2 mm/s (Sample A‐4) produced higher tensile strength. As shown in Table 3, the tensile properties of the weld samples varied significantly with the tool velocity ratio (Sample A‐3). At 2 mm/s, TiB_2_ (A‐4) produced a substantial UTS increase (238.6 MPa) versus unreinforced (205.15 MPa). Figure 9 depicts the stress‐strain and load‐displacement curves for Sample A‐5, where the ultimate tensile strength increased significantly, reaching 284.9 MPa under 3 mm/s traverse speed without TiB2. According to Table 5, the sample with TiB2 at 3 mm/s (Sample A‐5) exhibited the best ultimate tensile strength. At a velocity of 3 mm/s, the unreinforced specimen (A‐5) unexpectedly attained the maximum UTS of 284.9 MPa, exceeding that of the reinforced specimen (A‐6). As illustrated in Figure 10, the stress‐strain curve for Sample A‐6, processed with TiB2 at 3 mm/s, shows a moderate increase in tensile strength compared with its unreinforced counterpart. Table 6 shows the mechanical properties of the sample welded at 3 mm/s with TiB2 reinforcement (Sample A‐6), highlighting a significant increase in hardness. Figure 11 presents the stress‐strain curve and load‐displacement curves for Sample A‐7, processed at 4 mm/s without TiB2, indicating a slight improvement in tensile strength but lower elongation compared with the reinforced sample. The findings of Table 7 demonstrate the mechanical properties of the weld at 4 mm/s (Sample A‐7), where the unreinforced sample showed higher tensile strength compared with its reinforced counterpart. At a speed of 4 mm/s, TiB_2_ (A‐8) once again surpassed unreinforced (A‐7) in UTS (243.78 vs. 253.57 MPa) and elongation. TiB_2_ consistently improved ductility, particularly at low traverse rates, although ultimate tensile strength trends were influenced by speed. Optimal tensile characteristics were seen at intermediate speeds with reinforcement, but the peak UTS occurred at high speed without reinforcement, demonstrating complicated interaction between velocity ratio, heat input, and particle strengthening effects.

Figure 6Sample‐2: 1000 rpm/1 mm/s/1.5° with TiB2. (a) Stress–strain Curve A‐2. (b) Load–displacement Curve A‐2.

**Figure:**
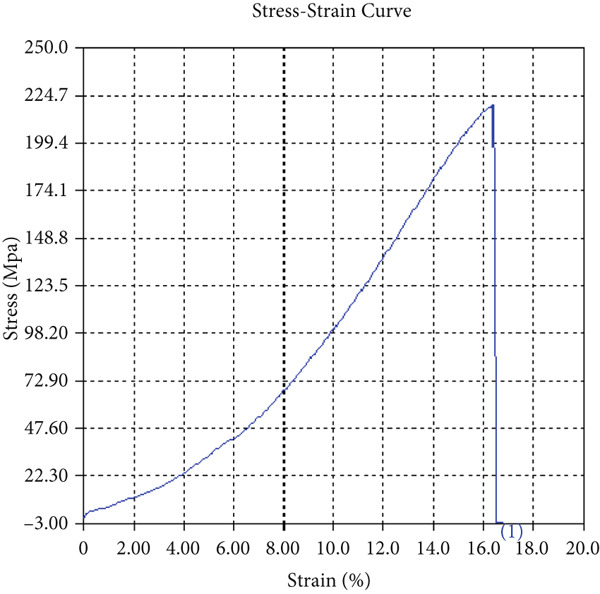
(a)

**Figure:**
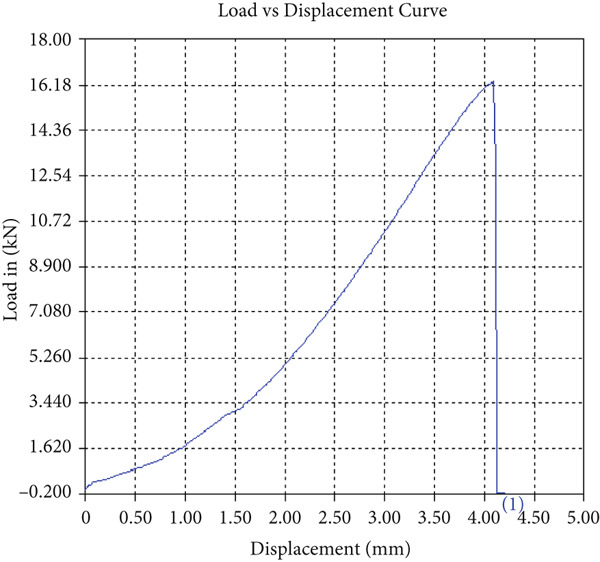
(b)

Figure 7Sample‐3: 1000 rpm/2 mm/s/1.5° without TiB2. (a) Stress–strain Curve A‐3. (b) Load–displacement Curve A‐3.

**Figure:**
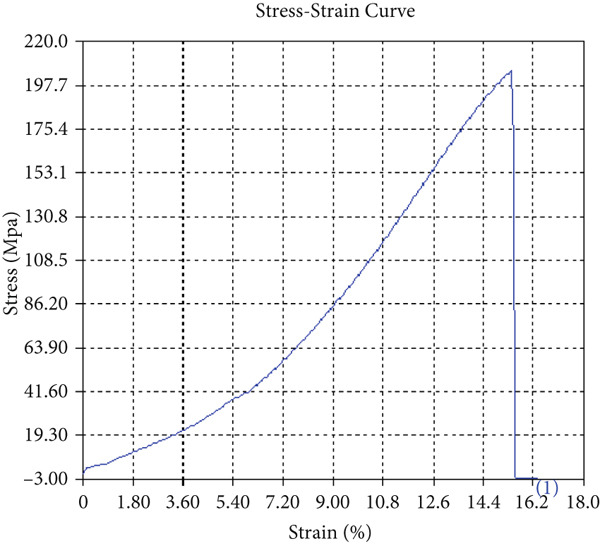
(a)

**Figure:**
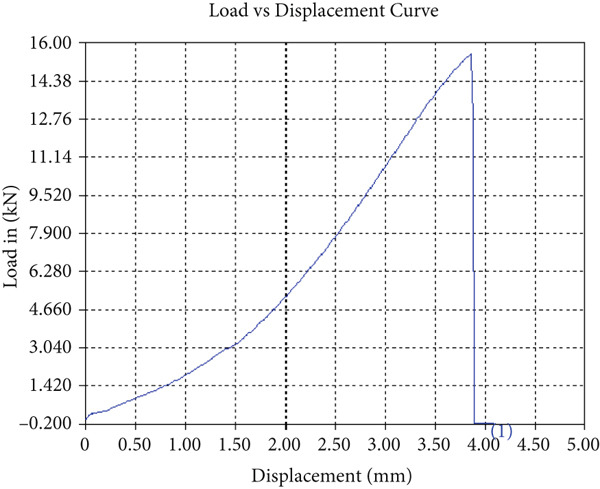
(b)

Figure 8Sample‐4: 1000 rpm/2 mm/s/1.5° with TiB2. (a) Stress–strain Curve A‐4. (b) Load–displacement Curve A‐4.

**Figure:**
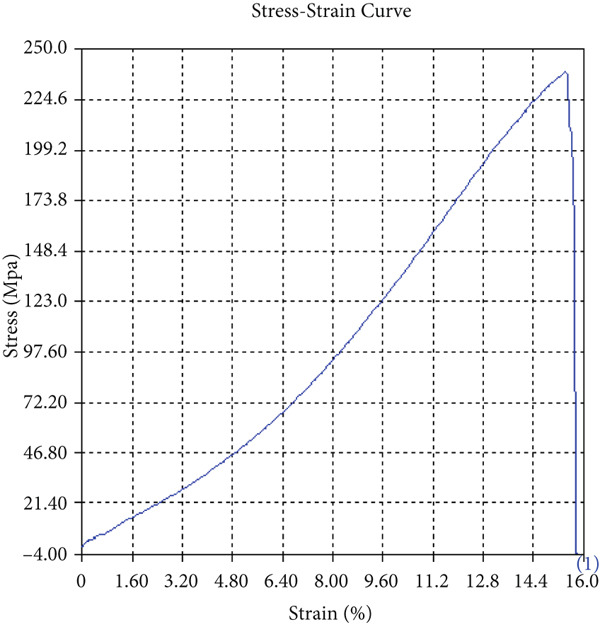
(a)

**Figure:**
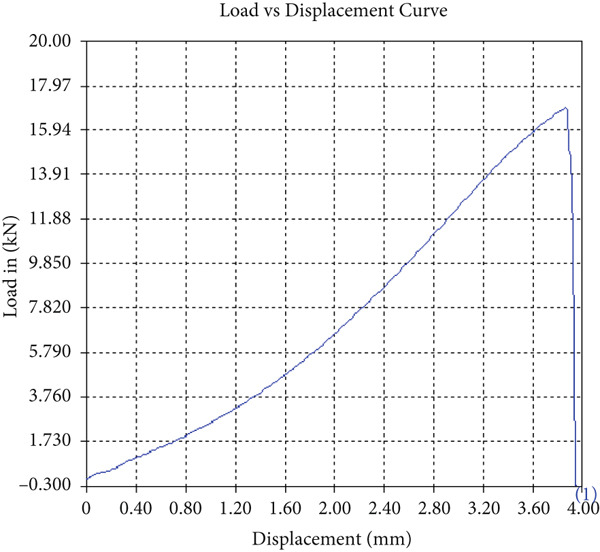
(b)

Figure 9Sample‐5: 1000 rpm/3 mm/s/1.5° without TiB2. (a) Stress–strain Curve A‐5. (b) Load–displacement Curve A‐5.

**Figure:**
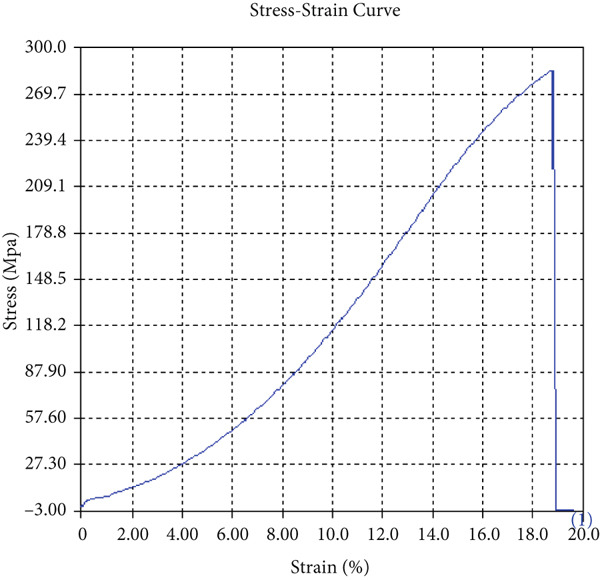
(a)

**Figure:**
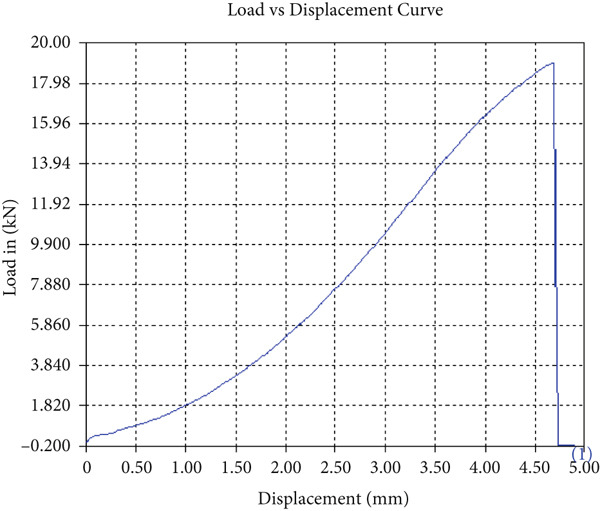
(b)

Figure 10Sample‐6: 1000 rpm/3 mm/s/1.5° with TiB2. (a) Stress–strain Curve A‐6. (b) Load–displacement Curve A‐6.

**Figure:**
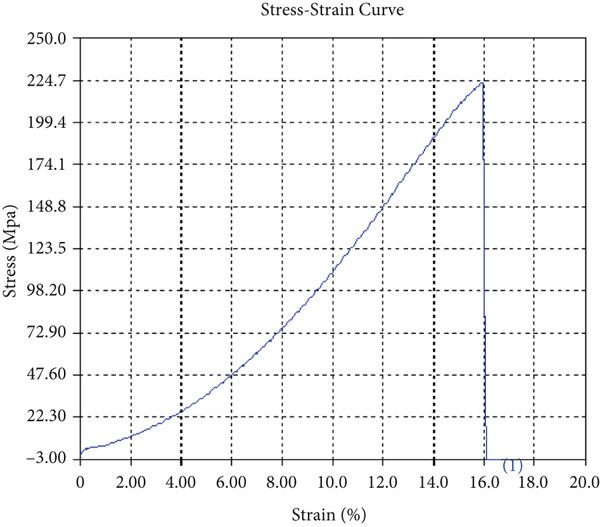
(a)

**Figure:**
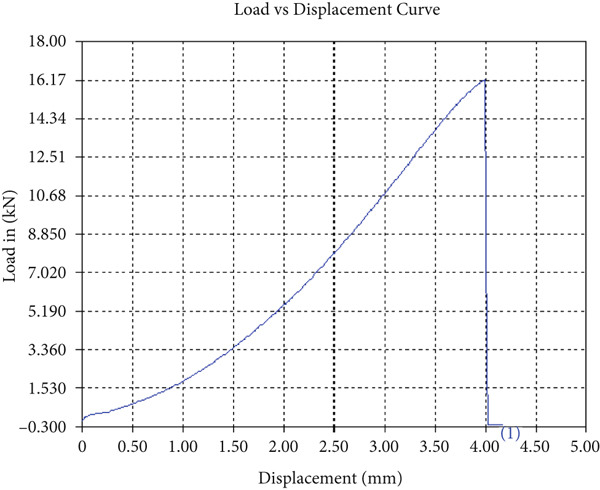
(b)

Figure 11Sample‐7: 1000 rpm/4 mm/s/1.5° without TiB2. (a) Stress–strain Curve A‐7. (b) Load–displacement Curve A‐7.

**Figure:**
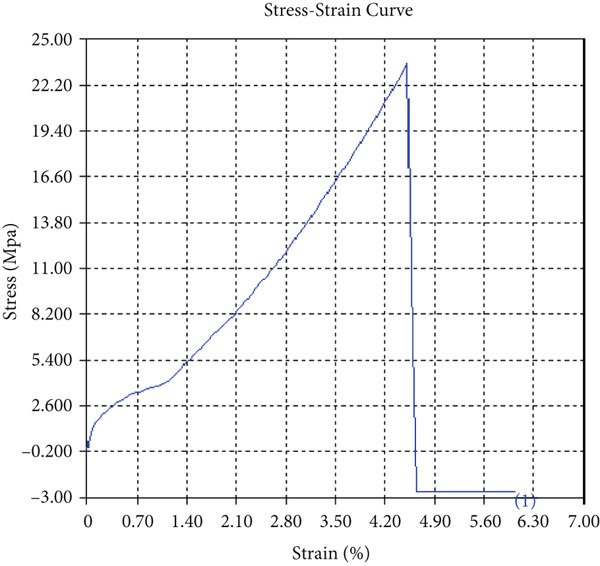
(a)

**Figure:**
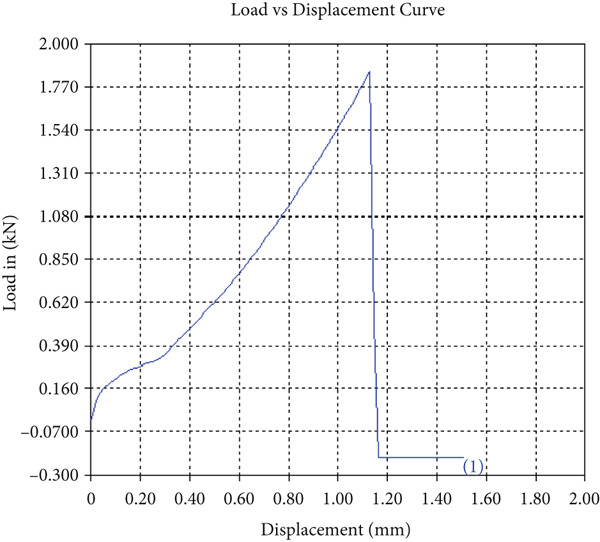
(b)

Figure 12Sample‐8: 1000 rpm/4 mm/s/1.5° with TiB2. (a) Stress–strain Curve A‐8. (b) Load–displacement Curve A‐8.

**Figure:**
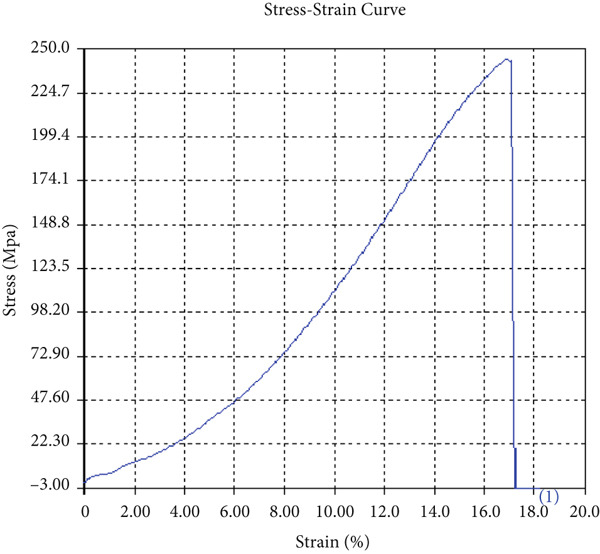
(a)

**Figure:**
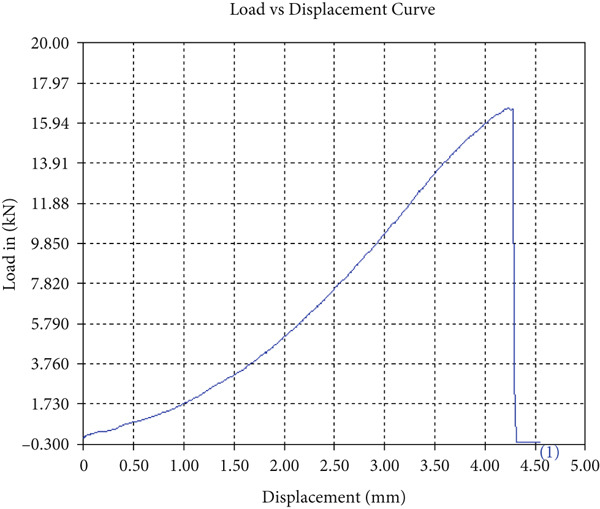
(b)

**Table 2:** Mechanical properties from tensile test of Sample A‐2.

Change in length (mm)	42.76
Maximum tensile force (kN)	16.33
Maximum yield force (kN)	12.99
Young′s modulus E (GPa)	1.44
Percentage of elongation (%)	6.9
Ultimate tensile stress (Mpa)	219.4892473
Maximum yield stress (Mpa)	174.5967742
0.2% Offset yield stress (Mpa)	178.0887097

**Table 3:** Mechanical Properties from Tensile Test of Sample A‐3.

Change in length (mm)	41.48
Maximum tensile force (kN)	15.53
Maximum yield force (kN)	12.73
Young′s modulus E (GPa)	1.39
Percentage of elongation (%)	3.7
Ultimate tensile stress (MPa)	205.1519155
Maximum yield stress (MPa)	168.1638045
0.2% Offset yield stress (MPa)	171.5270806

**Table 4:** Mechanical properties from tensile test of Sample A‐4.

Change in length (mm)	41.46
Maximum tensile force (kN)	16.95
Maximum yield Force (kN)	13.91
Young′s modulus E (GPa)	1.51
Percentage of elongation (%)	3.65
Ultimate tensile stress (MPa)	238.597973
Maximum yield stress (MPa)	195.8051802
0.2% Offset yield stress (MPa)	199.7212838

**Table 5:** Mechanical properties from tensile test of Sample A‐5.

Change in length (mm)	41.63
Maximum tensile force (kN)	19.02
Maximum yield force (kN)	14.25
Young′s modulus E (GPa)	1.67
Percentage of elongation (%)	4.075
Ultimate tensile stress (MPa)	284.9011384
Maximum yield stress (MPa)	213.4511684
0.2% Offset yield stress (MPa)	217.7201917

**Table 6:** Mechanical properties from tensile test of Sample A‐6.

Change in length (mm)	41.71
Maximum tensile force (kN)	16.2
Maximum yield force (kN)	12.5
Young′s modulus E (GPa)	1.44
Percentage of elongation (%)	4.275
Ultimate tensile stress (MPa)	223.2942798
Maximum yield Stress (MPa)	172.294969
0.2% Offset yield stress (MPa)	175.7408684

**Table 7:** Mechanical properties from tensile test of Sample A‐7.

Change in length (mm)	40.91
Maximum tensile force (kN)	16.86
Maximum yield force (kN)	12.31
Young′s modulus E (GPa)	1.45
Percentage of elongation (%)	2.275
Ultimate tensile stress (MPa)	253.57115701
Maximum yield stress (MPa)	166.60119123
0.2% Offset yield stress (MPa)	146.93321506

**Table 8:** Mechanical properties from tensile test of Sample A‐8.

Change in length (mm)	41.47
Maximum tensile force (kN)	16.67
Maximum yield force (kN)	12.63
Young’s modulus E (GPa)	1.54
Percentage of elongation (%)	3.675
Ultimate tensile stress (MPa)	243.7847324
Maximum yield stress (MPa)	184.7031296
0.2% Offset yield stress (MPa)	188.3971922

This improvement reflects better strain accommodation within the stirred zone, facilitated by a refined grain structure and more homogeneous microstructural distribution. The superior performance can be directly attributed to the enhanced material flow and heat input conditions associated with slower traverse speeds, which promote extensive plastic deformation and efficient thermal cycling. These thermal‐mechanical conditions contribute to better dispersion of the TiB_2_ particles within the weld nugget, resulting in grain refinement through pinning mechanisms and suppression of grain growth during cooling.

Furthermore, the presence of TiB_2_ particles acts as microstructural stabilizers, promoting load transfer and improving dislocation resistance within the matrix. These mechanisms are consistent with prior studies that emphasize the role of ceramic reinforcements and optimal process parameters in enhancing joint strength and integrity [27]. Thus, the findings underscore the synergistic effect of reinforcement and optimized tool velocity ratio in tailoring the tensile properties of dissimilar aluminum alloy weldments.

### 4.2. Hardness Distribution

**Figure 13:**
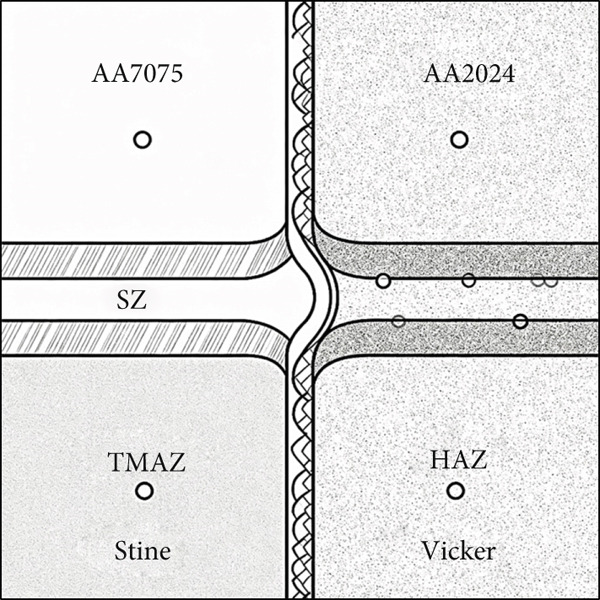
Schematic diagram of hardness taking location.

Microhardness profiles measured across the different weld zones—including the stir zone (SZ), thermo‐mechanically affected zone (TMAZ), and heat‐affected zone (HAZ)—demonstrated a marked improvement in hardness values for the TiB_2_‐reinforced friction stir‐welded joints compared with their unreinforced counterparts. Specifically, the peak hardness in the nugget (stir) zone increased from approximately 80 HV in unreinforced joints to around 95 HV in reinforced specimens, particularly at lower traverse speeds (e.g., 1 mm/s). Table 9 gives us the hardness values. This enhancement can be primarily attributed to the dispersion strengthening mechanism provided by the uniformly distributed TiB_2_ ceramic particles. These hard reinforcements act as barriers to dislocation motion and effectively hinder grain boundary sliding, thereby improving hardness. Additionally, the presence of TiB_2_ promotes dynamic recrystallization during the stirring process, leading to significant grain refinement in the NZ—another contributor to the increased hardness via the Hall–Petch effect. A schematic diagram has been added in Figure 13 showing the exact indentation locations across the SZ, TMAZ, and HAZ for hardness testing.

**Table 9:** Hardness test results.

**Sample**	**Parameters**	**Hardness values**
A‐1	1000 rpm/1 mm/s/1.5° without TiB_2_	118.25
A‐2	1000 rpm/1 mm/s/1.5° with TiB_2_	137.04
A‐3	1000 rpm/2 mm/s/1.5° without TiB_2_	154.11
A‐4	1000 rpm/2 mm/s/1.5° with TiB_2_	161.68
A‐5	1000 rpm/3 mm/s/1.5° without TiB_2_	172.52
A‐6	1000 rpm/3 mm/s/1.5° with TiB_2_	174.28
A‐7	1000 rpm/4 mm/s/1.5° without TiB_2_	167.08
A‐8	1000 rpm/4 mm/s/1.5° with TiB_2_	166.69

However, an inverse relationship was observed between traverse speed and microhardness values in the NZ. At higher traverse speeds, the reduction in heat input leads to inadequate plastic flow and poor mixing of the reinforcement within the aluminum matrix. This nonuniform dispersion of TiB_2_ particles reduces the effectiveness of the strengthening mechanisms. The tool velocity ratio (ratio of rotational speed to traverse speed) was found to be a critical parameter in optimizing the microstructural uniformity and mechanical performance of the joint. A higher velocity ratio ensures sufficient heat generation and stirring action, enhancing the metallurgical bonding and uniform dispersion of reinforcements, which in turn results in superior hardness characteristics across the weldment.

### 4.3. Uncertainty Analysis

The mechanical properties reported in this study are accompanied by an uncertainty analysis to reflect measurement variability and experimental reproducibility. Uncertainties were estimated as ± 3% of the average values of elongation, UTS, yield strength, Young′s modulus, and hardness based on typical precision limits of the testing methods employed. Table 10 summarizes the mechanical property values along with their corresponding uncertainty estimates, represented as error bars in the figures. Including these uncertainty ranges provides a clearer understanding of the confidence in the reported data and highlights the consistency of results across different welding conditions. This approach supports the reliability of the observed trends related to the influence of tool velocity ratio and TiB_2_ reinforcement on joint performance.

**Table 10:** Uncertainty analysis of mechanical properties for friction stir‐welded alloy joints.

**Sample**	**Elongation (%)**	**Elongation uncertainty (%)**	**UTS (MPa)**	**UTS uncertainty (MPa)**	**Yield strength (MPa)**	**Yield strength uncertainty (MPa)**	**Young′s modulus (GPa)**	**Young′s modulus uncertainty (GPa)**	**Hardness (HV)**	**Hardness uncertainty (HV)**
A‐1	4.375	0.131	217.10	6.513	179.16	5.375	1.41	0.042	118.25	3.548
A‐2	6.900	0.207	219.50	6.585	174.60	5.238	1.44	0.043	137.04	4.111
A‐3	3.700	0.111	205.15	6.154	168.16	5.045	1.39	0.042	154.11	4.623
A‐4	3.650	0.110	238.60	7.158	195.80	5.874	1.51	0.045	161.68	4.850
A‐5	4.080	0.122	284.90	8.547	213.45	6.403	1.67	0.050	172.52	5.176
A‐6	4.280	0.128	223.30	6.699	172.29	5.169	1.44	0.043	174.28	5.229
A‐7	2.280	0.068	253.57	7.607	166.60	4.998	1.45	0.044	167.08	5.012
A‐8	3.680	0.110	243.78	7.313	184.70	5.541	1.54	0.046	166.69	5.001

### 4.4. Microstructural Observations

Optical and scanning electron microscopy analyses revealed significant differences in the microstructural evolution of the NZ with respect to traverse speed and the presence of TiB_2_ reinforcement. Figure 14 deliberates the microstructural observations of the test specimens. At lower traverse speeds (e.g., 1 mm/s), the samples exhibited well‐defined, equiaxed, and ultrafine grains within the NZ, characteristic of dynamic recrystallization (DRX). This is attributed to the increased tool velocity ratio (rotational speed to traverse speed), which facilitates prolonged thermal exposure and intense plastic deformation—both of which are essential for DRX. In the TiB_2_‐reinforced samples, the grains were even finer and more uniformly distributed. This microstructural refinement is a direct consequence of particle‐stimulated nucleation (PSN) mechanisms and Zener pinning effects. The TiB_2_ particles act as nucleation sites for new grains and effectively hinder grain boundary migration during cooling, thereby restricting grain coarsening.

Figure 14Microstructure observation with and without TiB_2_ at 100× and 500× zoom in.

**Figure:**
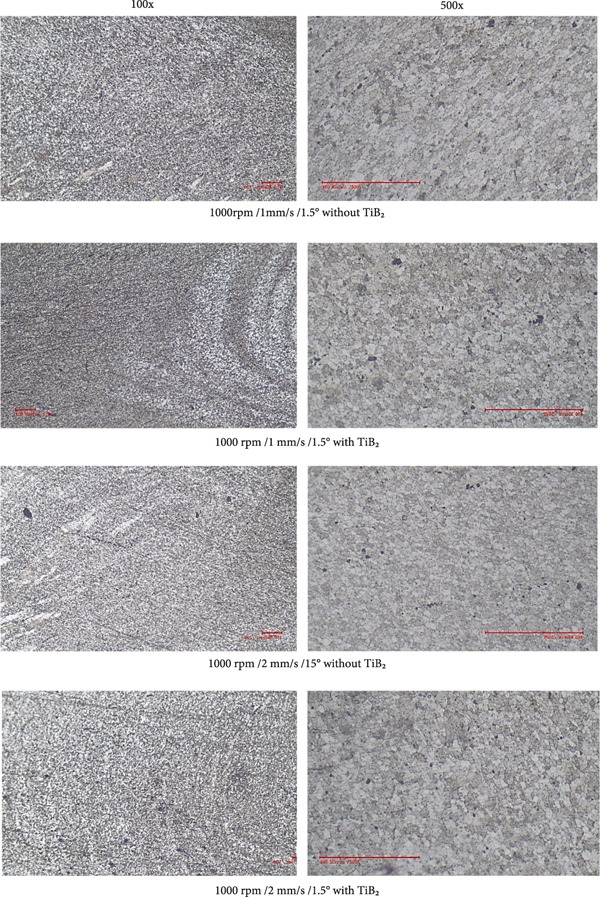
(a)

**Figure:**
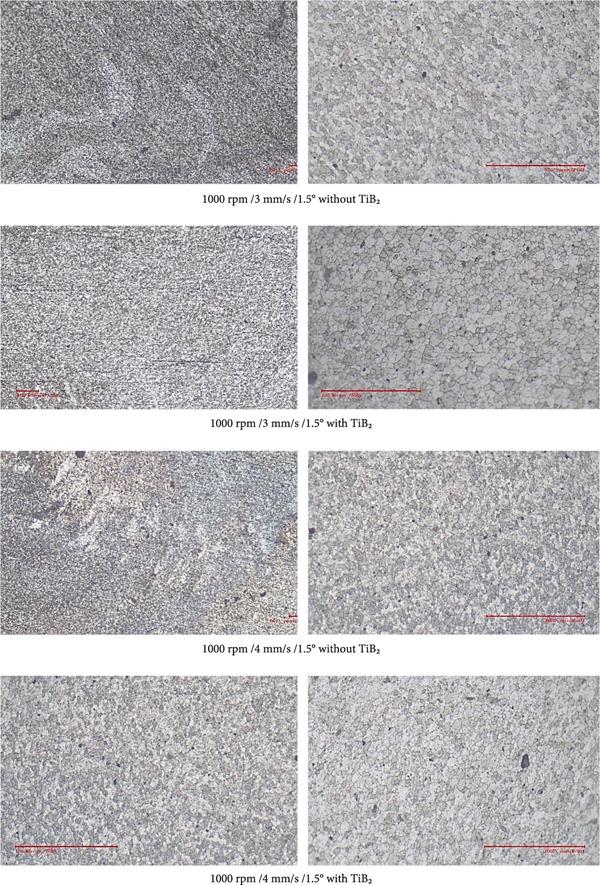
(b)

Conversely, unreinforced joints showed relatively coarser grains and, in some cases, nonuniform microstructures, especially at higher traverse speeds (3–4 mm/s). At these higher speeds, the interaction time between the tool and material is reduced, leading to insufficient plastic flow, decreased heat input, and incomplete dynamic recrystallization. This results in poor metallurgical bonding and the formation of weld defects such as tunnel voids and kissing bonds. The findings corroborate previous studies [28], emphasizing that optimum heat input—achieved through a balanced tool velocity ratio—is essential for uniform material flow, effective dispersion of reinforcements, and consistent grain refinement across the stir zone. Therefore, both reinforcement and process parameter optimization are pivotal in achieving defect‐free welds with superior microstructural integrity.

### 4.5. Effect of Tool Velocity Ratio

The tool velocity ratio (*ω*/v), defined as the ratio of the rotational speed (*ω*) to the traverse speed (v) of the FSW tool, plays a pivotal role in governing the heat generation, material flow behavour, and consequently, the overall quality of the weld. At a high velocity ratio—for example, 1000 rpm rotational speed with a traverse speed of 1 mm/s (*ω*/v = 1000)—an optimal thermal cycle is achieved, promoting intense plastic deformation and sufficient dwell time for effective stirring and dispersion of reinforcement particles such as TiB_2_. Under these conditions, the welds demonstrated superior mechanical properties, including the highest observed UTS and microhardness in the NZ. The enhanced performance is attributed to uniform microstructural evolution, improved metallurgical bonding, and refined grain structures due to both dynamic recrystallization and particle‐induced effects.

However, as the traverse speed increased to 4 mm/s (resulting in a velocity ratio of 250), a significant reduction in heat input per unit length occurred. This caused incomplete plasticization, inadequate mixing, and nonuniform particle distribution within the matrix, which degraded the weld quality. Although TiB_2_‐reinforced joints consistently outperformed their unreinforced counterparts across all welding conditions, the magnitude of improvement diminished at lower velocity ratios due to insufficient energy input and poor consolidation. These findings align with the thermal–mechanical interaction models of FSW and reinforce the necessity of optimizing the tool velocity ratio. An adequately high *ω*/v ensures sustained heat generation and mechanical stirring, both of which are essential for homogeneous material flow, defect‐free joints, and maximized mechanical performance [29].

### 4.6. Comparative Evaluation

A comprehensive comparison of all eight friction stir‐welded samples—comprising both TiB_2_‐reinforced and unreinforced joints across various traverse speeds—revealed that the incorporation of TiB_2_ ceramic particles yields the most pronounced mechanical benefits at lower traverse speeds (e.g., 1–2 mm/s). Under these conditions, the prolonged interaction between the rotating tool and the workpiece ensures adequate thermal input and extensive plastic deformation, which are critical for the uniform dispersion of reinforcement particles. This leads to an enhanced load transfer mechanism, superior particle‐matrix bonding, and more effective grain refinement via dynamic recrystallization. The TiB_2_ particles serve as potent nucleation sites during grain formation and act as barriers to grain boundary movement, a phenomenon known as Zener pinning. Additionally, the dispersion strengthening effect inhibits dislocation mobility, thereby contributing to increased hardness and strength.

However, as the traverse speed increases to 3–4 mm/s, the reduced dwell time and lower heat generation result in suboptimal mixing and uneven distribution of TiB_2_ particles, diminishing the effectiveness of the reinforcement. This trend is evident from the mechanical performance metrics, where the differential between reinforced and unreinforced joints narrows at higher speeds.

Figures illustrate this clearly, showing improved tensile strength and elongation in the TiB_2_‐reinforced samples under low‐speed conditions. The stress–strain curves exhibit higher peak loads and greater ductility, indicating enhanced energy absorption capacity of the weld zone. These findings align with earlier research involving Al_2_O_3_ and SiC particle reinforcements, which similarly demonstrated improvements in mechanical properties due to microstructural refinement and interfacial strengthening mechanisms [30]. Overall, this comparison highlights that the mechanical advantage of ceramic reinforcement is highly sensitive to processing parameters, particularly the tool traverse speed, and is optimized at lower velocity regimes.

## 5. Conclusion

This study conclusively demonstrates that both the tool velocity ratio (*ω*/v) and the incorporation of TiB_2_ ceramic particles are critical in enhancing the mechanical and microstructural performance of friction stir‐welded dissimilar joints between AA7075 and AA2024 aluminum alloys. The optimal welding condition was identified at a traverse speed of 1 mm/s (velocity ratio = 1000), under which the reinforced joint achieved a maximum tensile strength of 219.5 MPa and an elongation of 6.9%. The presence of TiB_2_ contributed to significant grain refinement through dynamic recrystallization and PSN, along with increased hardness via dispersion strengthening and grain boundary pinning. These improvements were most effective under low‐speed, high‐heat‐input conditions, ensuring uniform particle distribution and superior metallurgical bonding. In contrast, higher traverse speeds resulted in diminished reinforcement effectiveness due to reduced thermal exposure and mixing. The findings highlight the sensitivity of mechanical properties to process parameters and underscore the value of ceramic reinforcements in tailoring weld zone characteristics. Future investigations may focus on the use of nano‐scale reinforcements, hybrid particle systems, and in situ thermal characterization techniques to achieve further refinement and real‐time process control for optimized weld integrity and performance. However, the present study also has certain limitations. The experiments were conducted under a fixed tool rotational speed of 1000 rpm, and therefore the influence of higher or variable rotational speeds on heat generation and microstructural evolution was not explored. Additionally, the reinforcement particle size and distribution were not quantitatively analyzed using advanced imaging or mapping techniques such as EDS or EBSD. The study was restricted to TiB_2_ reinforcement; thus, hybrid or nano‐reinforced systems may provide different strengthening mechanisms. Furthermore, thermal measurements during welding were not recorded, which limits the ability to precisely correlate heat input with mechanical outcomes.

Future work should therefore focus on real‐time thermal–mechanical monitoring, optimization across a broader parameter window, and evaluation of long‐term performance aspects such as fatigue and corrosion resistance of the reinforced joints.

## 6. Contribution to Knowledge

The present study contributes to the growing body of knowledge on FSW of dissimilar aluminum alloys, particularly AA7075 and AA2024, in the following ways:

• **Novel correlation between tool velocity ratio and reinforcement behavior:** This work establishes a clear relationship between the tool velocity ratio (*ω*/v) and the mechanical, as well as the microstructural performance of TiB_2_‐reinforced dissimilar aluminum joints, which have not been comprehensively reported before.
• **Optimization of process parameters for enhanced weld quality:** The study demonstrates that a high velocity ratio (*ω*/v = 1000) significantly improves tensile strength, hardness, and ductility by promoting optimal heat input, uniform particle dispersion, and dynamic recrystallization.
• **Microstructural insights on reinforcement dispersion:** Detailed microstructural analysis revealed the role of TiB_2_ particles in Zener pinning and PSN mechanisms, providing a deeper understanding of grain refinement in hybrid FSW joints.
• **Industrial relevance for lightweight structures:** The results offer practical guidance for aerospace and automotive industries by demonstrating that TiB_2_‐reinforced FSW joints at optimized velocity ratios can yield high‐strength, defect‐free, and lightweight structural components.
• **Foundation for future research:** The findings create a benchmark for future studies on nano‐ or hybrid reinforcement systems, encouraging further exploration into in situ process monitoring and advanced characterization of thermomechanical behavior in FSW.

## Consent

Consent for publication was obtained from all participants whose data are included in this article. All identifying information has been anonymized to protect participant confidentiality.

## Disclosure

Each author has reviewed the final version of the manuscript and approved it for publication. No other individual who contributed to this work has been omitted without consent. All authors agree to be accountable for the integrity and accuracy of the research presented in this manuscript. No third‐party services were taken for this manuscript.

## Conflicts of Interest

The authors declare no conflicts of interest.

## Author Contributions

Mutyala Rama Durga Rao, Bunga Kiran Kumar: writing, investigation, data collection. Kiran Kumar Billa: supervision, methodology. Abhijit Bhowmik, N. Ashok: funding, figure preparation, writing review. All listed authors have made significant contributions to the conception, design, execution, or interpretation of the study.

## Funding

No funding was received for this manuscript.

## Data Availability

The data that support the findings of this study are available from the corresponding author upon reasonable request.
